# Supplementary material for: Prevalence and spatial distribution characteristics of human echinococcosis in China
Source: PLoS Negl Trop Dis. 2021 Dec 28;15(12):e0009996. doi: 10.1371/journal.pntd.0009996 (PMC8789093; doi:10.1371/journal.pntd.0009996)
Supplement: Attachment — Submitted filename: Comments.docx [file pntd.0009996.s001.docx]

Xiao-Nong Zhou and colleagues reported their original finding on prevalence and spatial distribution characteristics of human echinococcosis in China. This paper is informative, well written and may provide a thorough information regarding the prevalence and spatial distribution characteristics of human CE and AE in China. These date would be quite helpful for implementing prevention and control strategy for human echinococcosis and also is important for fighting against echinococcosis.

However, there are still some concerns regarding this paper:

1. Page4, Line 73, “For the year 2018, 47,233”. This presentation is quite misunderstanding, please correct;

2. Page 11, Line 190, “The number of CA cases……”. This should be CE please correct.

3. Page 11 Line 198. In this section, author stated there are still some unclassified cases. Would you please explain why in some counties have unclassified cases?

4. Page 13, Line 209 “In the national echinococcosis prevention and control” should be written as “National Echinococcosis Prevention and Control”.

5. Page 18, Line 250, “Further analyzed the prevalence of human CE and the prevalence of human AE ” . This sentence should be corrected and whole paper should be revised for English for better reading.

6.
