# Supplementary material for: Prevalence and spatial distribution characteristics of human echinococcosis in China
Source: PLoS Negl Trop Dis. 2021 Dec 28;15(12):e0009996. doi: 10.1371/journal.pntd.0009996 (PMC8789093; doi:10.1371/journal.pntd.0009996)
Supplement: Attachment — Submitted filename: Answer to reviewers.docx [file pntd.0009996.s002.docx]

**Answer to reviewers**

**Reviewer #1:**

**Comment 1. Page4, Line 73, “For the year 2018, 47,233”. This presentation is quite misunderstanding, please correct;**

**Answer:** The manuscript was modified accordingly. Please check: Page 4, line 80.

**Comment 2. Page 11, Line 190, “The number of CA cases……”. This should be CE please correct.**

**Answer:** The error has been corrected accordingly. Please check: Page 11, line 211.

**Comment 3. Page 11 Line 198. In this section, author stated there are still some unclassified cases. Would you please explain why in some counties have unclassified cases?**

**Answer:** Most of the reasons were that detailed classification records were ignored, and some of them were not sure whether they were cystic or vesicular. As the B-ultrasound pictures at that time were not saved, so there was no way to screen and reclassify. The manuscript was modified accordingly.

**Comment 4. Page 13, Line 209 “In the national echinococcosis prevention and control” should be written as “****National Echinococcosis Prevention and Control”.**

**Answer:** The manuscript was modified accordingly. Please check: Page 14, lines 235-236.

**Comment 5. Page 18, Line 250, “Further analyzed the prevalence of human CE and the prevalence of human AE ” . This sentence should be corrected and whole paper should be revised for English for better reading.**

**Answer:** Dear reviewer, thank you for this constructive comment. The sentence has been corrected. Please check: Page 19, lines 278. Also, as suggested, appropriate corrections have been made and the manuscript has been revised for English accordingly.

**Reviewer #2:**

**Comment 1. The MS is suggested to be** **reviewed by people whose native language is English.**

**Answer:** Dear reviewer, thank you for this constructive comment. As suggested, appropriate corrections have been made and the manuscript has been revised for English.

**Comment 2. In Abstract, the part of methods is too brief. For example, the distribution of 370 counties should be described. SPSS 21.0 and ArcGIS 10.1 were used to obtain the prevalence rate of AE and CE. Chi-square test and Exact probability method were used to get the message of ?......The overall situation or prevalence rate of echinococcosis in China should be presented in the part of results as well as in Conclusion.**

**Answer:** Thank you for your expert comments. The manuscript was modified accordingly, with appropriate clarifications and insertions made. Please check: Page 2, line 42-48.

**Comment 3. P5 line 103, Xinjiang should be replaced by Xinjiang Uygur Autonomous Region.**

**Answer:** The manuscript was modified accordingly. Please check: Page 5, line 112.

**Comment 4. P15 Table 2, please check the meaning of P, p value or prevalence rate?**

**Answer:** The meaning of ‘P’ in Table 2 has been provided. Please check.

**Comment 5. P16 Table3, the meaning of P should showed below the table as well as Table 4.**

**Answer:** The meaning of ‘P’ in Table 3 and Table 4 has been provided. Please check.

**Comment 6. P18 line 249-250, “the prevalence of human CE and the prevalence of human AE” can be replaced by “the prevalence of human CE and AE” as well as P24 line 309, “ways of production, way of life” to “ways of production, and life and religious…”**

**Answer:** The manuscript was modified accordingly. Please check: Page 19, lines 278; and Page 25, line 342.

**Comment 7. P25 line 324, “ownd dogs” should be “owned dogs”.**

**Answer:** The manuscript was modified accordingly. Please check: Page 27, line 374.

**Comment 8. P26 line 339-340 not only the gradient was west-east oriented but no north-south variation was observed. What the meaning?**

**Answer:** We apologize for this misunderstanding. In the process of draft writing, we tried to do the trend analysis of echinococcosis cases, but the results had no significance or value. We are sorry to forget to delete such a content before submitting the manuscript. It has been deleted in this revised version.

**Comment 9. In Discussion, the difference of prevalence rate and spatial distribution between AE and CE should be discussed and analyzed.**

**Answer:** Dear reviewer, thank you for this important comment. The manuscript has been revised accordingly. Please check: Page 26, line 345-361.Dogs are definitive hosts for *E. granulosus senso lato* and *E. multilocularis* in China [8,21]. *E. granulosus senso lato* and *E. multilocularis* have different intermediate hosts [21]. *Echinococcus* parasites need two mammalian hosts to complete their life cycle [7]. Transmission of *E. granulosus s.l.* occurs between dogs and livestock, while *E. multilocularis* transmission occurs between stray dogs or foxes as definitive hosts and rodents as intermediate hosts [21]. Humans are aberrant intermediate hosts and they are infected by ingesting *E. multilocularis* eggs [22]. The involvement of wildlife in *E. multilocularis* transmission cycle is not clear and further researches are needed to investigate this issue. However, the spatial distribution of the prevalence of human echinococcosis directly reflects the range and degree of the corresponding transmission cycle of CE and AE. It can thus guide prevention and control activities, such as strengthening the control of stray dogs in the epidemic areas of AE, carrying out control measures of intermediate host animal density and monitoring the infection status. Health education and people awareness should also be considered. According to the different epidemic degrees of CE and the local control capacity, the frequency of deworming should be determined to achieve the purpose of scientific guidance.
