# Supplementary material for: Prevalence and spatial distribution characteristics of human echinococcosis in China
Source: PLoS Negl Trop Dis. 2021 Dec 28;15(12):e0009996. doi: 10.1371/journal.pntd.0009996 (PMC8789093; doi:10.1371/journal.pntd.0009996)
Supplement: Attachment — Submitted filename: Answer to reviewers 2021-10-07.docx [file pntd.0009996.s003.docx]

**Answer to reviewers**

**Reviewer #1:**

**Comment 1.** **Page 4 Line 73, "renforcement" should be written as "reinforcement".**

**Answer:** The error has been corrected accordingly. Please check: Page 4, line 73.

**Comment 2. Page 5 Line 102 "They heavily impair the patients, especially AE, with a mortality rate of about 90% in the past ten years". Please confirm, the mortality is about 90% in AE without any intervention.**

**Answer:** Dear reviewer, thank you for this constructive comment. The sentence has been corrected. Please check: Page 5, line 102.

**Comment 3. Page 23 Line 303 "explaned" should be written as "explained".**

**Answer:** The error has been corrected accordingly. Please check: Page 23, line 309.
